# Cutaneous leishmaniasis mimicking pyoderma gangrenosum after traveling to Costa Rica

**DOI:** 10.1016/j.jdcr.2024.08.006

**Published:** 2024-09-21

**Authors:** Stephanie Waggett, Tammam Alanazi, Hank Kearse

**Affiliations:** a College of Medicine, Medical University of South Carolina, Charleston, South Carolina; b Department of Dermatology and Dermatologic Surgery, Medical University of South Carolina, Charleston, South Carolina

**Keywords:** cutaneous leishmaniasis, *Leishmania panamensis*, miltefosine, nonhealing ulcer

## Introduction

Leishmaniasis is a vector-borne disease, primarily originating in subtropical and tropical locations throughout the world. Cutaneous manifestations of leishmaniasis include ulcerating plaques commonly with erythematous satellite lesions as well as nodules that may rarely spread in a sporotrichoid pattern, as in this case. *Leishmania panamensis* is one species of Leishmania and generally responds to miltefosine or amphotericin B treatment. We present a case of cutaneous leishmaniasis in a patient with recent travel to Costa Rica who adequately responded to a 2-month course of miltefosine. Notably, he developed leishmaniasis-associated superficial venous thrombosis, a rare complication of leishmaniasis that has not been previously reported.

## Case report

**Fig 1:**
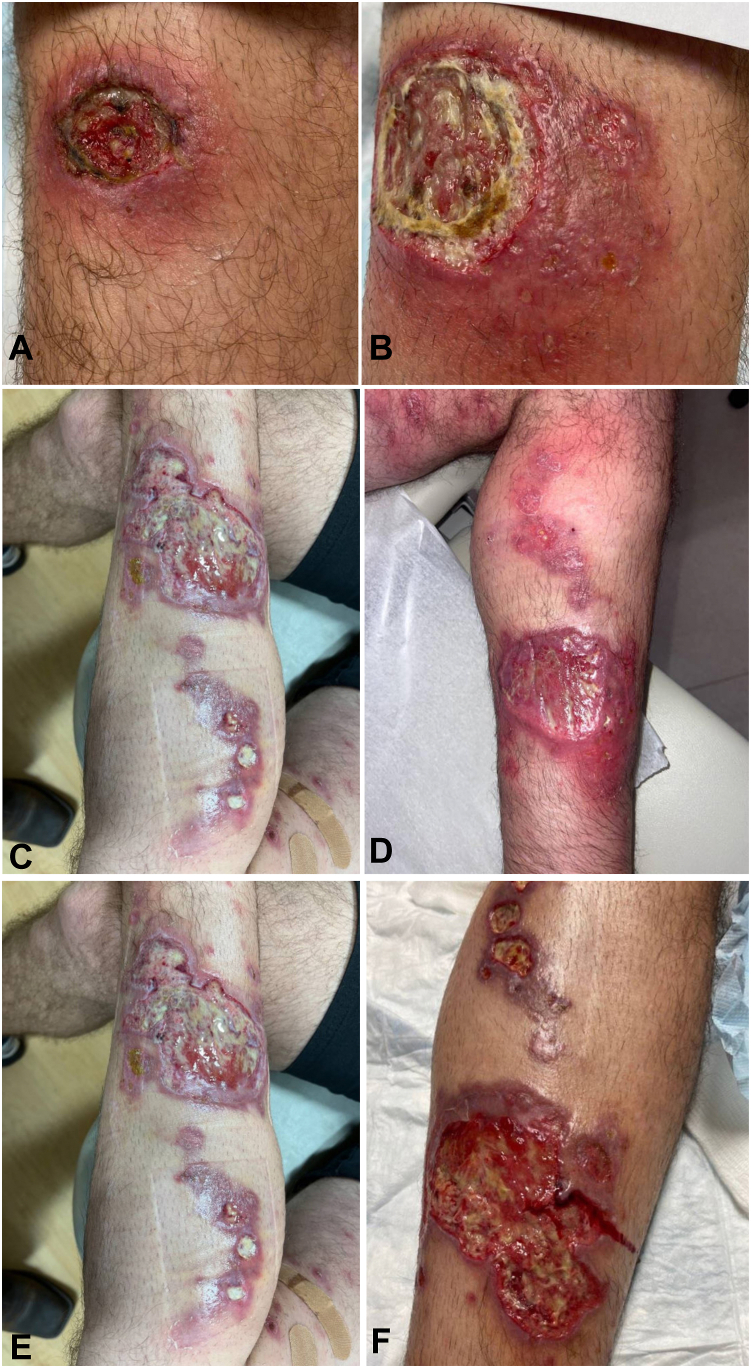
Clinical progression of the lesion, presenting as a localized ulcer (**A**) that progressively worsened (**B**, **C**) and spread in a sporotrichoid pattern (**D-F**) over the next several months.

A 47-year-old otherwise healthy male presented with a month-long left lower extremity nonhealing ulcer with initial concern for a spider bite. The lesion began as a small red papule which gradually progressed in size and started to ulcerate (Fig 1, *A*). He received 2 courses of antibiotics and underwent debridement of necrotic tissue.

**Fig 2:**
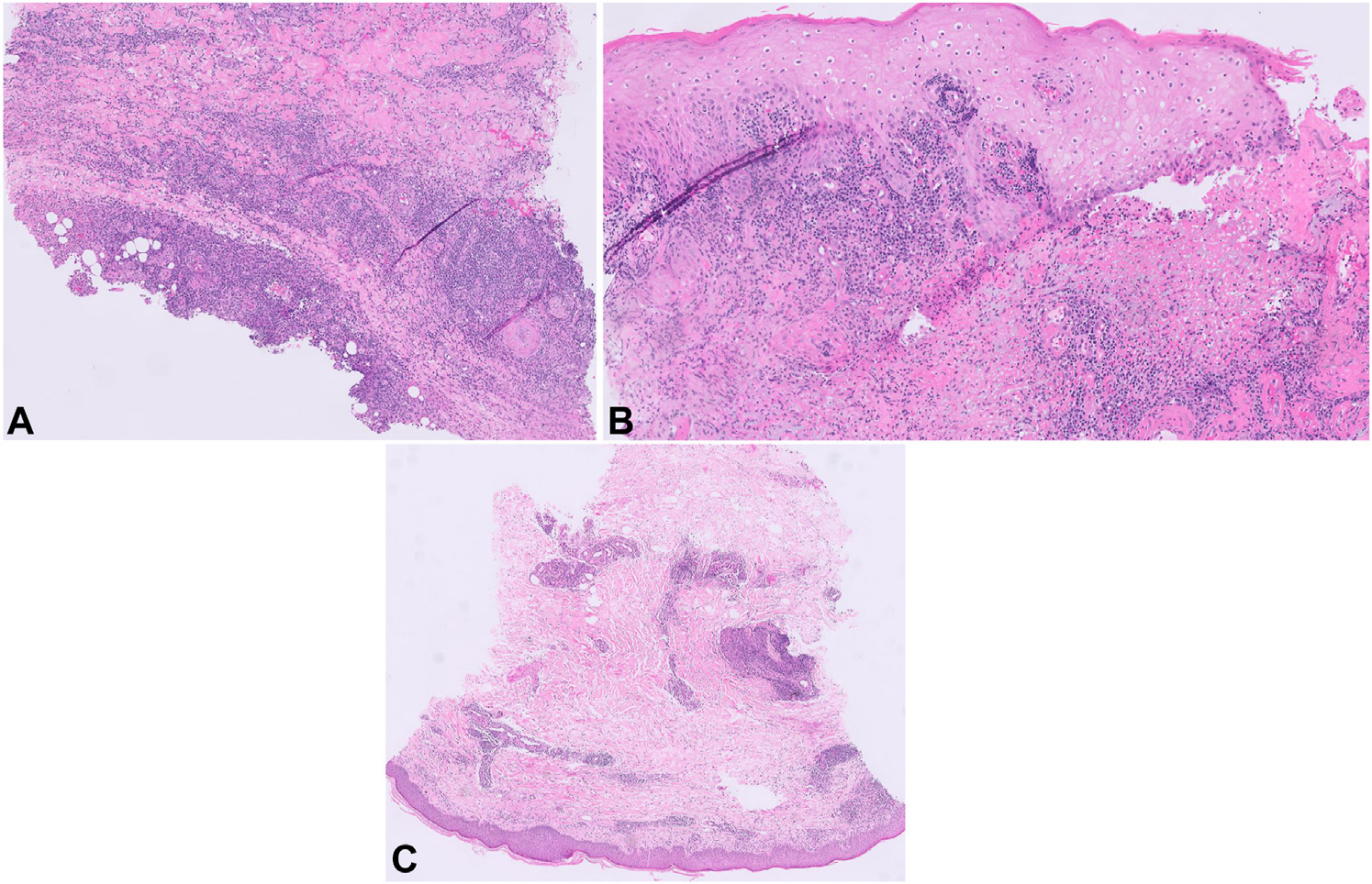
Histopathology of left leg punch biopsy. Initial biopsy at an outside facility showed pseudoepitheliomatous hyperplasia, inflammation, and necrosis, as seen at 5× (**A**) and 10× (**B**) power. Repeat biopsy about 3 weeks later revealed perivascular inflammation and rare intracellular organisms, shown at 2× (**C**) power.

He followed with wound care over the next month, though the lesion continued to worsen (Fig 1, *B*). He denied having any other cutaneous or mucosal symptoms. Bacterial tissue cultures were negative. While awaiting biopsy results, he received a course of high-dose steroids from his wound care physician for presumed diagnosis of pyoderma gangrenosum versus vasculitis. The 3-week tapered course subjectively provided him improvement. Left lower extremity Doppler showed superficial venous thrombosis proximal to the lesion, extending from the level of the left midthigh to the left mid-calf. Apixaban was subsequently initiated. Given the ulcer’s elevated violaceous border (Fig 1, *C*); improvement with steroids, clobetasol, and timolol; and negative laboratory workup (including comprehensive metabolic panel, antinuclear antigen, protein electrophoresis, and rheumatoid factor), dermatology started him on cyclosporine which was continued for 6 weeks. Four biopsies obtained at an outside institution revealed nonspecific pseudoepitheliomatous hyperplasia with inflammation and necrosis (however no neutrophils typical for pyoderma gangrenosum); acid-fast bacilli and periodic acid-Schiff (PAS) diastase stain were negative (Fig 2, *A* and *B*).

**Fig 3:**
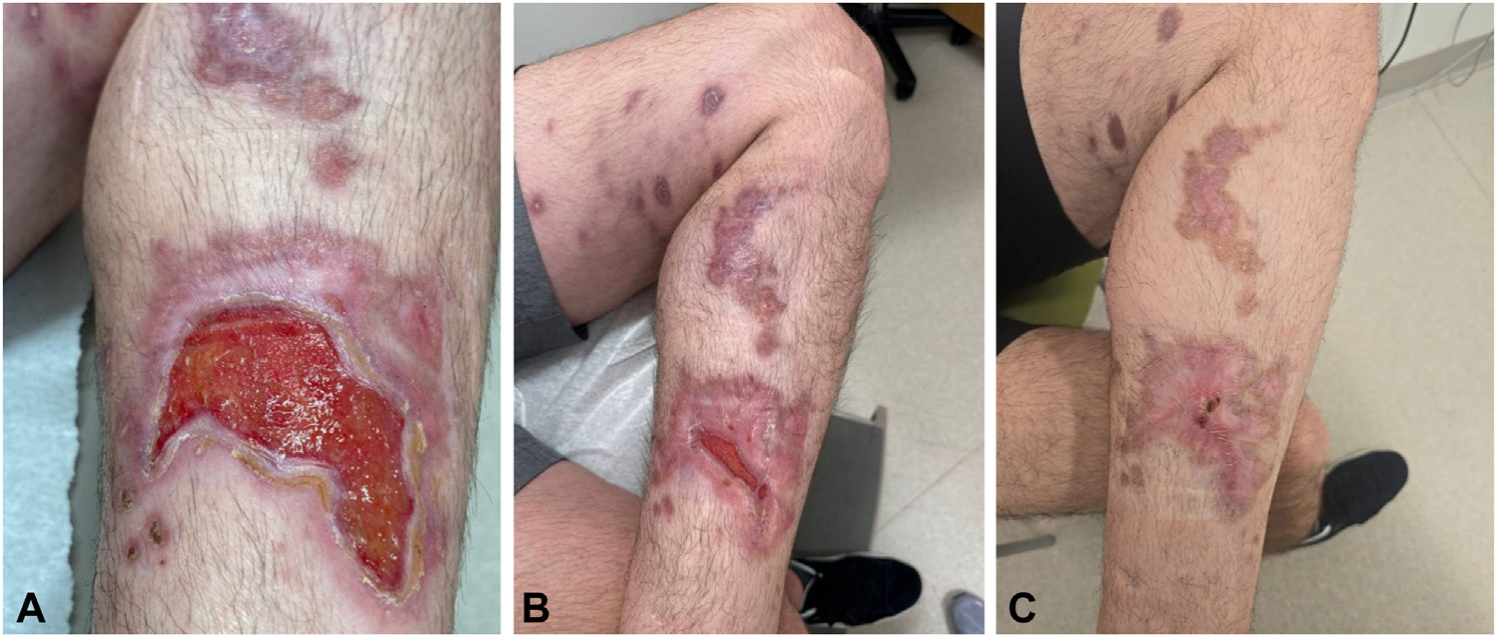
Healing of cutaneous leishmaniasis on miltefosine as shown after 1.5 months of treatment course (**A**) and at 3 months (**B**) and 6 months (**C**) after initiation of treatment. Patient was treated for a total of 2 months on miltefosine, then maintained on basic wound care.

The patient later developed worsening nodules along his leg in a sporotrichoid pattern (Fig 1, *D*-*F*). At that time, he noted that he had traveled to Costa Rica earlier that year, a few weeks before his initial presentation. Cyclosporine was discontinued, and he underwent repeat biopsies with concern for an infectious etiology including leishmaniasis and deep fungal infection. The biopsy showed superficial and deep perivascular inflammation with rare intracellular organisms (Fig 2, *C*). Giemsa stain and CD1a were negative for organisms. Thus, polymerase chain reaction was recommended for definitive diagnosis given the high suspicion. Polymerase chain reaction performed at the Centers for Disease Control and Prevention confirmed cutaneous *Leishmania panamensis*, and he was started on miltefosine 50 mg 3 times daily. The patient improved significantly on miltefosine, and evidence of healthy granulation tissue and wound closure was noted at follow-ups (Fig 3, *A*). Given his abrupt improvement in clinical status with the initiation of miltefosine after his prior decline, the source of his healing was likely treatment success from miltefosine versus natural resolution.

His treatment course with miltefosine was a total of 2 months. Subsequently, he continued to follow-up with wound care and dermatology and reported that he was able to exercise and resume normal activities. Physical exam revealed lower extremity post-inflammatory hyperpigmentation and scarring; however, the ulcerations showed significant healing (Fig 3, *B*). His visit 6 months after initiating miltefosine revealed near-complete lesion resolution (Fig 3, *C*).

## Discussion

There are 3 types of leishmaniasis: visceral, cutaneous, and mucosal, each arising from a sandfly bite.^1^ Cutaneous leishmaniasis presents as chronic, progressive, erythematous papules and plaques that tend to ulcerate.^1^ Certain species, including *Leishmania panamensis*, may involve the mucosa (though mucosal involvement was absent in our patient). Diagnosis is made via histopathology, with polymerase chain reaction available through the Centers for Disease Control and Prevention to delineate species. Miltefosine is Food and Drug Administration-approved for *Leishmania panamensis*, *Leishmania braziliensis*, and *Leishmania guyanensis*.^1^

Pavlidakey et al (2015) discussed a patient who developed *Leishmania panamensis* from a visit to Costa Rica, with lesions similarly presenting in a sporotrichoid pattern. This patient was successfully treated with IV sodium stibogluconate, another indicated treatment for this species.^2^ Achtman et al (2016), Ergen and King (2015), and Mann et al (2020) also reported a total of 5 patients who developed cutaneous *Leishmania panamensis* from trips to Costa Rica, who were treated, respectively, with amphotericin B,^3^ stibogluconate,^4^ and miltefosine.^5^ Vélez et al (1994) discussed a patient who developed *Leishmania panamensis* in Colombia, with an initial presentation similar to our patient: a local papule that significantly progressed in size and cutaneous involvement. This patient was successfully treated with another first-line therapy, meglumine antimoniate.^6^ Superficial venous thrombosis associated with leishmaniasis is not widely reported. Pathophysiology of thrombosis formation in this case likely stems from damage to the venous wall secondary to the infection and ulceration.

This case highlights the importance of a thorough travel history and the broad differential diagnosis of cutaneous ulcers. This case also serves as a reminder that pyoderma gangrenosum is a diagnosis of exclusion, and all potential causes, especially infectious, should be ruled out prior to systemic immunosuppression. Fortunately, this patient received timely treatment once he was diagnosed with *Leishmania panamensis* and ultimately experienced healing. Dermatologists should keep cutaneous leishmaniasis on the differential in cases of unresolving ulcerative lesions, as cases of leishmaniasis are not uncommon in the United States. Furthermore, this case highlights a unique manifestation of cutaneous leishmaniasis—superficial venous thrombosis—that clinicians should monitor for when treating patients with this condition.

## Conflicts of interest

None disclosed.
